# Impact of COVID-19 on mental health and quality of life: Is there any effect? A cross-sectional study of the MENA region

**DOI:** 10.1371/journal.pone.0249107

**Published:** 2021-03-25

**Authors:** Ayesha S. Al Dhaheri, Mo’ath F. Bataineh, Maysm N. Mohamad, Abir Ajab, Amina Al Marzouqi, Amjad H. Jarrar, Carla Habib-Mourad, Dima O. Abu Jamous, Habiba I. Ali, Haleama Al Sabbah, Hayder Hasan, Lily Stojanovska, Mona Hashim, Osama A. Abd Elhameed, Reyad R. Shaker Obaid, Samar ElFeky, Sheima T. Saleh, Tareq M. Osaili, Leila Cheikh Ismail

**Affiliations:** 1 Department of Nutrition and Health, College of Medicine and Health Sciences, United Arab Emirates University, Al Ain, United Arab Emirates; 2 Department of Sport Rehabilitation, Faculty of Physical Education and Sport Sciences, The Hashemite University, Zarqa, Jordan; 3 Department of Clinical Nutrition and Dietetics, College of Health Sciences, University of Sharjah, Sharjah, United Arab Emirates; 4 Research Institute of Medical and Health Sciences, University of Sharjah, Sharjah, United Arab Emirates; 5 Department of Health Services Administration, College of Health Sciences, University of Sharjah, Sharjah, United Arab Emirates; 6 Faculty of Agricultural and Food Sciences, Nutrition Department, American University of Beirut, Riad El-Solh, Beirut, Lebanon; 7 College of Natural and Health Sciences, Zayed University, Dubai, United Arab Emirates; 8 Institute for Health and Sport, Victoria University, Melbourne, Victoria, Australia; 9 Nutrition and Dietetics Program, School of Health Sciences, Universiti Sains Malaysia, Kubang Kerian, Kelantan, Malaysia; 10 Port Fouad Hospital, Egyptian Ministry of Health, Port Said, Egypt; 11 Community-Based Initiatives and Health for Older People, World Health Organization, Regional Office for Eastern Mediterranean Region, Cairo, Egypt; 12 Department of Nutrition and Food Technology, Faculty of Agriculture, Jordan University of Science and Technology, Irbid, Jordan; 13 Nuffield Department of Women’s & Reproductive Health, University of Oxford, Oxford, United Kingdom; Population Council, INDIA

## Abstract

The COVID-19 pandemic is a major health crisis that has changed the life of millions globally. The purpose of this study was to assess the effect of the pandemic on mental health and quality of life among the general population in the Middle East and North Africa (MENA) region. A total of 6142 adults from eighteen countries within the MENA region completed an online questionnaire between May and June 2020. Psychological impact was assessed using the Impact of Event Scale-Revised (IES-R) and the social and family support impact was assessed with questions from the Perceived Support Scale (PSS). The IES-R mean score was 29.3 (SD = 14.8), corresponding to mild stressful impact with 30.9% reporting severe psychological impact. Most participants (45%–62%) felt horrified, apprehensive, or helpless due to COVID-19. Furthermore, over 40% reported increased stress from work and financial matters. Higher IES-R scores were found among females, participants aged 26–35 years, those with lower educational level, and participants residing in the North Africa region (*p*<0.005). About 42% reported receiving increased support from family members, 40.5% were paying more attention to their mental health, and over 40% reported spending more time resting since the pandemic started. The COVID-19 pandemic was associated with mild psychological impact while it also encouraged some positive impact on family support and mental health awareness among adults in the MENA region. Clinical interventions targeted towards vulnerable groups such as females and younger adults are needed.

## Introduction

The novel coronavirus, later designated as COVID-19, is an infectious disease that can spread among humans. It emerged initially in the city of Wuhan in China in late December 2019, when cases of pneumonia of unknown etiology were reported [1]. Following its emergence, it manifested as an outbreak that led to serious public health concerns by the World Health Organization (WHO), and by mid-March 2020, the WHO declared a global pandemic due to the substantial global-wide spread of the disease affecting many countries [2]. By 14 February 2021, over 108 million cases were confirmed worldwide, of which 5.99 million cases were reported in the Eastern Mediterranean region [3].

In response to this global health crisis, quarantine and lock down measures were implemented by international and government health organizations to contain the rapid spread of the virus. Further measures included suspension of flights, avoidance of large gatherings, mandatory use of face mask in many countries, social distancing, teleworking, home-schooling of children and health orders to stay at home [4]. While the WHO and worldwide health authorities are actively working on containing the outbreak, such a period of health crisis has significant repercussions on human health and welling, accompanied by psychological distress and related symptoms such as stress, panic and anxiety in the general population [5]. Moreover, psychological impact is considered to be more profound in comparison to the Severe Acute Respiratory Syndrome (SARS) epidemic in 2003, due to the extensive social media exposure and increased global connectivity [6, 7]. SARS-related psychological problems have been reported to be prevalent mainly among healthcare workers and SARS survivors [8, 9]. In 2012, the Middle East respiratory syndrome coronavirus (MERS-CoV) was first identified in Saudi Arabia [10]. The spread of MERS-CoV across the Middle East was linked to the transmission of the pathogen from Dromedary camels to humans [11]. The MERS-CoV outbreak was associated with tremendous public anxiety in the affected countries, and it resulted in thousands of mortality cases, fear, anxiety, and psychosocial stress among the population, in addition to economic losses [12, 13]. Consequently, it is crucial to understand the extent of impact for such pandemics on mental health and other aspects of life [14, 15].

Historically, quarantine has been a successful measure adopted worldwide in infectious diseases outbreaks; however, it represents an unfavorable experience for the population. Movement restriction, separation from family or friends, limited freedom and fear of an uncertain future are all factors that may exacerbate negative psychological impact [16]. Large scale outbreaks as previously seen in the SARS epidemic have been associated with higher prevalence of psychological symptoms, emotional disturbance, depression, stress, post-traumatic stress symptoms and irritability [8]. Similarly, healthcare workers taking care of patients during the MERS-CoV outbreak in Saudi Arabia, reported feeling afraid and nervous, mainly about their safety as well as about colleagues and family, and the availability of infection control guidelines [17]. Literature shows that multiple stressors including longer duration of quarantine, fear of infection, distress, loneliness, boredom, confinement, inadequate information and financial loss, play a role in aggravating poor mental health [18].

The Middle East and North Africa (MENA) region in general, is a very sensitive area economically, politically, culturally and religiously. There are many challenges to contain the spread of COVID-19 in the region including political conflicts, humanitarian crises, suboptimal transparency, and frequent social and religious mass gatherings [19]. Additionally, the ongoing outbreak and the social isolation could have a huge impact on the mental health of individuals in the MENA region. Limited data is available on how people within the MENA region are coping with the COVID-19 pandemic and the extent of its ramifications on their mental health and well-being. Thus, this study aims to examine the impact of the COVID-19 outbreak on the mental health and quality of life among residents of the MENA region. The authors hypothesized that changes in family and social support, lifestyle changes, and increases in negative indicators were associated with higher IES-R total scores.

## Methods

### 1. Study design and participants

A cross-sectional, web-based survey was conducted in the MENA region between 11 May 2020 and 15 June 2020. The sample was drawn from eighteen countries within the MENA region; including Algeria, Bahrain, Egypt, Iraq, Jordan, Kuwait, Lebanon, Libya, Morocco, Oman, Palestine, Qatar, Republic of Yemen, Saudi Arabia, Sudan, Syria, Tunisia, and United Arab Emirates. Consenting adults aging 18 years and older were recruited electronically using convenience and snowball sampling methods in order to guarantee a large-scale distribution and recruitment of participants. There was no restriction on the total number of participants, however a minimum target of 100 participants from each country was desired. A total of 6142 participants (32.7% males) completed the survey and their data were included in the analysis.

The psychological impact of COVID-19 among adults was measured on the Impact of Event Scale-Revised (IES-R) and the social and family support impact was assessed using questions from the Perceived Support Scale (PSS) [20–22]. The questionnaires were prepared using Google Document Forms in the English, Arabic and French languages and it was automatically hosted via a unique URL. The electronic survey was pilot tested for clarity in a sample of 26 people from three countries in the MENA region. A few adjustments to wording were made preceding the pilot test to ensure the questionnaire’s clarity and applicability. A uniform resource locator (URL) was retrieved for the survey and was distributed formally (using e-mail invitations) and informally (using social media platforms, e.g., LinkedIn^™^, Facebook^™^, and WhatsApp^™^). In addition, researchers involved in this project distributed the survey to their contacts and work colleagues.

An information sheet and a consent form were available on the first page of the questionnaire in all three languages. Participants were free to withdraw at any time without giving explanations and no personal identification was requested to retain information confidentiality. Participants were given no incentives for participating in the questionnaire. The system of Google Forms only provides responses for questionnaires with 100% completion rate. The responses were download as an Excel file and securely stored using a password protected “Cloud” database. The present study followed the ethical code for web-based research [23, 24] and conforms to the principles embodied in the Declaration of Helsinki [25]. The study protocol was approved by the Social Sciences Research Ethics Committee at United Arab Emirates University (ERS_2020_6115).

### 2. Survey questionnaire

Information on the socio-demographic characteristics of the respondents was collected including age, gender, country of residence, education level, employment status, marital status, and work or study setting.

#### 2.1 Impact of Event Scale-Revised Scoring (IES-R)

The Impact of Event Scale-Revised (IES-R) was used to assess the psychological impact of COVID-19 among adults residing in the MENA region [20, 26]. The IES-R is a self-administered questionnaire containing 22 items and it has been previously translated and validated in the English, Arabic and French languages [27–30]. The IES-R has also been used to measure symptomatology experienced during the COVID-19 pandemic in Saudi Arabia, Egypt, Italy, and China [15, 22, 31–33]. The response for each question was scored based on a five-point Likert scale ranging from 0 (not at all) to 4 (extremely) and generated a total score (ranging from 0 to 88). The total IES-R score was considered normal (from 0 to 23); indicative of mild (from 24 to 32); moderate (from 33 to 36); or severe (≥ 37) psychological impact [15]. Three subscale scores were also calculated measuring intrusion (8 items), avoidance (8 items), and hyperarousal (6 items) [21].

#### 2.2 Indicators of negative mental health impact

Six modified and validated questions were asked regarding negative mental health impacts resulting from the COVID-19 pandemic [21]. Three questions inquired whether participants felt horrified, apprehensive, or helpless due to the pandemic. The other three questions assessed changes in stress from work, financial stress, and stress from home during the pandemic. The response options to these questions were much increased, increased, same as before, decreased, and much decreased.

#### 2.3 Impact on social and family support

This section included modified and validated questions from the Perceived Support Scale (PSS) assessing the impact of the COVID-19 pandemic on the support received from family or friends [15, 21]. It included five items including support from friends, support from family members, sharing feelings with a family member, sharing feelings with others when blue, and caring for family members’ feelings. The response options were much increased, increased, same as before, decreased, and much decreased.

#### 2.4 Mental health-related lifestyle changes

Participants were also asked to rate whether they were paying less or more attention to mental health related lifestyle changes during the COVID-19 pandemic using modified and validated questions [21]. This section included four items; attention to mental health, devoting enough time to rest, to relaxation, and to exercise. The response options were much increased, increased, same as before, decreased, and much decreased.

### 3. Statistical analysis

Normality of data was tested with the use of Kolmogorov-Smirnov test of normality. Descriptive statistics for the sociodemographic characteristics were reported as numbers and percentages. The IES-R total and subscale scores were presented as Median and Interquartile Range (IQR). A Chi-square (χ^2^) test was used to determine the association between IES-R categories (normal, mild, moderate, and severe) with categorical variables. A non-parametric Kruskal-Wallis H test was used to determine differences in IES-R, intrusion, avoidance, and hyperarousal scores between different regions. Followed by post-hoc pairwise comparisons with Bonferroni adjustment. A generalized linear model based on negative binomial distribution was used to assess the confounding effects of sociodemographic factors, negative mental health impact factors, social and family support indicators, and lifestyle factors on continuous IES-R total score. Variables included in the final model were selected using univariant general linear analysis and only factors with a cut-off value of *p*<0.2 were selected. A *p*-value <0.05 was considered to be statistically significant. Statistical analysis was performed using the Statistical Package for the Social Sciences (SPSS) version 26.0 (IBM, Chicago, IL, USA). The minimum sample size (n = 3246) was calculated using G*power software, version 3.1.9.4 (HHU, Germany) to detect a small effect size (0.10), with a power of 0.99, and alpha 0.05.

## Results

### 1. Demographic characteristics

The percentage of participants that completed the survey in the Arabic, English and French languages was 86.4%, 10.2% and 3.3% respectively. The sociodemographic characteristics of the study population are presented in Table 1. The female to male ratio was almost 2:1, with 32.7% males. The majority of surveyed individuals were aged 36–45 years (27.3%), were married (60.8%), had a bachelor’s degree (49.3%), worked full-time (44.5%), were working or studying from home (52.6%), and were residing in the Gulf region (48.7%).

**Table 1:** Sociodemographic characteristics of participants (*n* = 6142).

Variables	n (%)
Gender	
Females	4134 (67.3)
Males	2008 (32.7)
Age (years)	
18–25	1421 (23.1)
26–35	1512 (24.6)
36–45	1677 (27.3)
>46	1532 (24.9)
Marital status	
Married	3732 (60.8)
Single	2097 (34.1)
Divorced	234 (3.8)
Widowed	79 (1.3)
Education level	
School/diploma	1505 (24.5)
Bachelor’s degree	3026 (49.3)
Graduate degree	1611 (26.2)
Employment status	
Full-time	3657 (59.5)
Part-time	903 (14.7)
Unemployed	1582 (25.8)
Working/ studying from home	
Yes	3230 (52.6)
No	2416 (39.3)
Not applicable	496 (8.1)
Region of residence	
Gulf region^1^	2991 (48.7)
Levant region^2^	1448 (23.6)
North Africa region^3^	1703 (27.7)

^1^Gulf region: Bahrain, Kuwait, Qatar, Saudi Arabia, United Arab Emirates, Oman, and Republic of Yemen;

^2^Levant region: Jordan, Lebanon, Iraq, Syria, Palestine;

^3^North Africa region: Egypt, Algeria, Libya, Morocco, Sudan, and Tunisia

### 2. Impact of Event Scale-Revised Scoring (IES-R)

The overall mean IES-R score was 29.3 ± 14.8 (range 0–84), reflecting a mild stressful impact of the COVID-19 pandemic on the surveyed participants. The overall mean scores for the intrusion, avoidance and hyperarousal scales in participants were 9.5 ± 6.1, 11.8 ± 5.8, and 7.9 ± 5.0, respectively. A Kruskal-Wallis H analysis revealed an expected significant difference in total IES-R scores between the different regions, (*X*^2^ (2) = 102.937, *p*<0.001), with a mean rank IES-R score of 2897.95 for Gulf region, 3036.45 for Levant region, and 3471.21 for North Africa region (Table 2). Furthermore, there was a significant difference in intrusion scores between the different regions, (*X*^2^ (2) = 106.650, *p*<0.001), with a mean rank intrusion score of 2913.00 for Gulf region, 2996.70 for Levant region, and 3486.87 for North Africa region. A significant difference in avoidance scores between regions were observed, (*X*^2^ (2) = 38.410, *p*<0.001), with a mean rank avoidance score of 2984.50 for Gulf region, 3010.56 for Levant region, and 3322.88 for North Africa region. Moreover, there was a significant difference in hyperarousal scores between the different regions, (*X*^2^ (2) = 109.964, *p*<0.001), with a mean rank hyperarousal score of 2853.84 for Gulf region, 3142.34 for Levant region, and 3437.78 for North Africa region.

**Table 2:** Psychological impact of COVID-19 on participants by region (*n* = 6142).

Variables	All (*n* = 6142)	Gulf region^1^ (*n* = 2991)	Levant region^2^ (*n* = 1448)	North Africa region^3^ (*n* = 1703)	p value*
IES-R, Median (IQR)					
Total score	28 (18–39)	27 (17–37) ^a^ ^Ŧ^	28 (18–38) ^b^	32 (22–43) ^c^	<0.001
Intrusion	9 (5–14)	8 (4–13) ^a^	8 (5–13) ^a^	11 (6–16) ^b^	<0.001
Avoidance	12 (8–16)	11 (7–15) ^a^	11 (7–16) ^a^	13 (9–17) ^b^	<0.001
Hyperarousal	7 (4–11)	7 (3–10) ^a^	8 (4–11) ^b^	9 (5–13) ^c^	<0.001

IES-R: Impact of Event Scale–Revised; IQR: Interquartile Range

*p-value was based on Kruskal-Wallis H test.

^Ŧ^Values with different superscript letters are significantly different, based on pairwise comparisons with Bonferroni adjustment (*p*<0.05).

^1^Gulf region: Bahrain, Kuwait, Qatar, Saudi Arabia, United Arab Emirates, Oman, and Republic of Yemen;

^2^Levant region: Jordan, Lebanon, Iraq, Syria, Palestine;

^3^North Africa region: Egypt, Algeria, Libya, Morocco, Sudan, and Tunisia

### 3. Sociodemographic and Impact Event Scale-Revised (IES-R)

The association of IES-R scores with sociodemographic factors is presented in Table 3. A Chi-square analysis revealed significant association between IES-R categories with gender (*X*^2^ = 36.440; *p*<0.001), age (*X*^2^ = 54.585; *p*<0.001), education level (*X*^2^ = 32.663; *p*<0.001), employment status (*X*^2^ = 65.989; *p* = 0.017), and region of residence (*X*^2^ = 102.244; *p*<0.001). As expected, the multivariate regression analysis revealed that females (Estimated rate ratio: 9.1%; *p* = 0.003), participants aged 26–35 years (Estimated rate ratio: 12.2%; *p* = 0.022), school or college diploma graduates (Estimated rate ratio: 9.4%; *p* = 0.034), and residence of North Africa region (Estimated rate ratio: 11.8%; *p*<0.001) were more likely to have higher IES-R scores. However, marital status, employment status and working from home were not significantly associated with changes in total IES-R score.

**Table 3:** Association of IES-R scores with sociodemographic factors (*n* = 6142).

Variables	All	IES-R categories				*P*-value*	Rate Ratio (CI 95%)	*P*-value**
	*n* = 6142	Normal	Mild	Moderate	Severe			
		n (%)	n (%)	n (%)	n (%)			
		*n* =	*n* =	*n* =	*n* =			
Gender								
Females	4134 (67.3)	1466 (63.1)	932 (68.0)	377 (68.4)	1359 (71.7)	<0.001	1.091 (1.030–1.155)	0.003
Males	2008 (32.7)	859 (36.9)	439 (32.0)	174 (31.6)	536 (28.3)		1	
Age (years)								
18–25	1421 (23.1)	532 (22.9)	291 (21.2)	125 (22.7)	473 (25.0)	<0.001	1.104 (0.998–1.221)	0.022
26–35	1512 (24.6)	522 (22.5)	338 (24.7)	138 (25.0)	514 (27.1)		1.122 (1.038–1.213)	
36–45	1677 (27.3)	598 (25.7)	393 (28.7)	160 (29.0)	526 (27.8)		1.095 (1.019–1.177)	
>46	1532 (24.9)	673 (28.9)	349 (25.5)	128 (23.2)	382 (20.2)		1	
Marital status								
Married	3732 (60.8)	1401 (60.3)	870 (63.5)	337 (61.2)	1124 (59.3)	0.171	0.830 (0.660–1.044)	0.212
Single	2097 (34.1)	806 (34.7)	444 (32.4)	178 (32.3)	669 (35.3)		0.789 (0.621–1.003)	
Divorced	234 (3.8)	93 (4.0)	42 (3.1)	28 (5.1)	71 (3.7)		0.846 (0.652–1.098)	
Widowed	79 (1.3)	25 (1.1)	15 (1.1)	8 (1.5)	31 (1.6)		1	
Education level								
School/diploma	1505 (24.5)	524 (22.5)	352 (25.7)	134 (24.3)	495 (26.1)	<0.001	1.094 (1.013–1.181)	0.034
Bachelor’s degree	3026 (49.3)	1109 (47.7)	659 (48.1)	294 (53.4)	964 (50.9)		1.083 (1.014–1.156)	
Graduate degree	1611 (26.2)	692 (29.8)	360 (26.3)	123 (22.3)	436 (23.0)		1	
Employment status								
Full-time	3657 (59.5)	1435 (61.7)	786 (57.3)	330 (59.9)	1106 (58.4)	0.017	1.026 (0.958–1.100)	0.362
Part-time	903 (14.7)	296 (12.7)	212 (15.5)	84 (15.2)	311 (16.4)		1.066 (0.976–1.165)	
Unemployed	1582 (25.8)	594 (25.5)	373 (27.2)	137 (24.9)	478 (25.2)		1	
Working/Studying from home								
Yes	3230 (52.6)	1253 (53.9)	687 (50.1)	297 (53.9)	993 (52.4)	0.202	1.024 (0.926–1.133)	0.833
No	2416 (39.3)	882 (37.9)	559 (40.8)	218 (39.6)	757 (39.9)		1.008 (0.913–1.113)	
Not applicable	496 (8.1)	190 (8.2)	125 (9.1)	36 (6.5)	145 (7.7)		1	
Region of residence								
Gulf region^1^	2991 (48.7)	1246 (53.6)	683 (49.8)	253 (45.9)	809 (42.7)	<0.001	0.949 (0.892–1.010)	<0.001
North Africa region^2^	1703 (27.7)	421 (18.1)	316 (23.0)	127 (23.0)	584 (30.8)		1.118 (1.040–1.203)	
Levant region^3^	1448 (23.6)	658 (28.3)	372 (27.1)	171 (31.0)	502 (26.5)		1	

IES-R: Impact of Event Scale–Revised; CI: confidence interval

*p-value was based on Chi-square test

** *p*-value was based on generalized linear model analysis

^1^Gulf region: Bahrain, Kuwait, Qatar, Saudi Arabia, United Arab Emirates, Oman, and Republic of Yemen;

^2^North Africa region: Egypt, Algeria, Libya, Morocco, Sudan, and Tunisia;

^3^Levant region: Jordan, Lebanon, Iraq, Syria, Palestine

### 4. Indicators of negative mental health impact

Table 4 presented the association between IES-R scores and negative mental health indicators. About 40% of the participants reported increased stress from work during the outbreak, 45.3% felt an increased level of stress from financial matters, and 60.3% of participants had increased stress from home during the pandemic. Furthermore, 61.0% of the participants felt horrified, 61.5% felt apprehensive, and 45.2% felt helpless due to the pandemic. A Chi-square analysis revealed significant association between IES-R categories with increased stress from work (*X*^2^ = 387.901; *p*<0.001), increased stress from financial matters (*X*^2^ = 5197.234; *p*<0.001), increased stress from home (*X*^2^ = 354.400; *p*<0.001), feeling horrified (*X*^2^ = 678.749; *p*<0.001), feeling apprehensive (*X*^2^ = 529.160; *p*<0.001), and feeling helpless (*X*^2^ = 496.914; *p*<0.001). The multivariate regression analysis detected that increased stress from work (Estimated rate ratio: 16.6%; *p*<0.001), increased financial stress (Estimated rate ratio: 6.3%; *p* = 0.027), increased stress from home (Estimated rate ratio: 10.9%; *p*<0.001), feeling horrified (Estimated rate ratio: 23.4%; *p*<0.001), feeling apprehensive (Estimated rate ratio: 9.3%; *p* = 0.008), and feeling helpless (Estimated rate ratio: 14.1%; *p*<0.001) were significantly associated with higher IES-R scores.

**Table 4:** Association of IES-R scores with negative mental health indicators (*n* = 6142).

Variables	All	IES-R categories				*P*-value*	Rate Ratio (CI 95%)	*P*-value**
	n (%)							
	*n* = 6142	Normal	Mild	Moderate	Severe			
		n (%)	n (%)	n (%)	n (%)			
		*n* = 2325	*n* = 1371	*n* = 551	*n* = 1895			
Increased stress from work								
No	3632 (59.1)	1693 (72.8)	817 (59.6)	308 (55.9)	814 (43.0)	<0.001	1	<0.001
Yes	2510 (40.9)	632 (27.2)	554 (40.4)	243 (44.1)	1081 (57.0)		1.166 (1.104–1.232)	
Increased Financial Stress								
No	3360 (54.7)	1507 (64.8)	748 (54.6)	284 (51.5)	821 (43.3)	<0.001	1	0.027
Yes	2782 (45.3)	818 (35.2)	623 (45.4)	267 (48.5)	1074 (56.7)		1.063 (1.007–1.123)	
Increased stress from home								
No	2439 (39.7)	1253 (53.9)	502 (36.6)	190 (34.5)	494 (26.1)	<0.001	1	<0.001
Yes	3703 (60.3)	1072 (46.1)	869 (63.4)	361 (65.5)	1401 (73.9)		1.109 (1.047–1.175)	
Felt horrified due to COVID-19								
No	2394 (39.0)	1357 (58.4)	487 (35.5)	174 (31.6)	376 (19.8)	<0.001	1	<0.001
Yes	3748 (61.0)	968 (41.6)	884 (64.5)	377 (68.4)	1519 (80.2)		1.234 (1.156–1.318)	
Felt apprehensive due to COVID-19								
No	2362 (38.5)	1296 (55.7)	473 (34.5)	176 (31.9)	417 (22.0)	<0.001	1	0.008
Yes	3780 (61.5)	1029 (44.3)	898 (65.5)	375 (68.1)	1478 (78.0)		1.093 (1.023–1.168)	
Felt helpless due to COVID-19								
No	3367 (54.8)	1647 (70.8)	752 (54.9)	271 (49.2)	697 (36.8)	<0.001	1	<0.001
Yes	2775 (45.2)	678 (29.2)	619 (45.1)	280 (50.8)	1198 (63.2)		1.141 (1.077–1.208)	

Answers of “much increased” and “increased” have been merged as “Yes”; Answers of “same as before”, “decreased” and “much decreased” have been merged as “No”; IES-R: Impact of Event Scale–Revised; CI: confidence interval;

*p-value was based on Chi-square test;

** *p*-value was based on generalized linear model analysis.

### 5. Impact on social and family support

Table 5 showed the association between IES-R score and family and social support. The results showed that 42.1% of the participants reported receiving increased support from family members, 24.3% received increases support from friends, and 48.1% stated increased shared feelings with their family members during the pandemic. In addition, the majority of participants (67.4%) cared more about their family members’ feelings following the onset of the pandemic.

**Table 5:** Association of IES-R scores with impact on family and social support (n = 6142).

Variables	All	IES-R categories				*P*-value*	Rate Ratio (CI 95%)	*P*-value**
	n (%)							
	*n* = 6142	Normal	Mild	Moderate	Severe			
		n (%)	n (%)	n (%)	n (%)			
		*n* = 2325	*n* = 1371	*n* = 551	*n* = 1895			
Getting support from friends								
Decreased	1223 (19.9)	429 (18.5)	266 (19.4)	112 (20.0)	416 (22.0)	<0.001	1	0.001
Same as before	3424 (55.7)	1539 (66.2)	771 (56.2)	291 (52.8)	823 (43.4)		0.943 (0.875–1.016)	
Increased	1495 (24.3)	357 (15.4)	334 (24.4)	148 (26.9)	656 (34.6)		1.083 (0.992–1.181)	
Getting support from family members								
Decreased	647 (10.5)	200 (8.6)	131 (9.6)	55 (10.0)	261 (13.8)	<0.001	1	0.201
Same as before	2907 (47.3)	1355 (58.3)	622 (45.4)	242 (43.9)	688 (36.3)		0.928 (0.838–1027)	
	2588 (42.1)	770 (33.1)	618 (45.1)	254 (46.1)	946 (49.9)		0.973 (0.876–1.080)	
Shared feelings with family members								
Decreased	748 (12.2)	210 (9.0)	164 (12.0)	72 (13.1)	302 (15.9)	<0.001	1	0.055
Same as before	2440 (39.7)	1246 (53.6)	510 (37.2)	176 (31.9)	508 (26.8)		0.846 (0.77–0.929)	
Increased	2954 (48.1)	869 (37.4)	697 (50.8)	303 (55.0)	1085 (57.3)		0.944 (0.857–1.039)	
Shared feelings with other when in blue								
Decreased	1388 (22.6)	437 (18.8)	316 (23.0)	142 (25.8)	493 (22.6)	<0.001	1	<0.001
Same as before	3065 (49.9)	1492 (64.2)	698 (50.9)	248 (45.0)	627 (33.1)		0.869 (0.809–0.933)	
Increased	1689 (27.5)	396 (17.0)	357 (26.0)	161 (29.2)	775 (40.9)		1.079 (0.996–1.169)	
Caring for family members’ feelings								
Decreased	256 (4.2)	91 (3.9)	47 (3.4)	16 (2.6)	102 (5.4)	<0.001	1	0.36
Same as before	1744 (28.4)	889 (38.2)	337 (24.6)	116 (21.1)	402 (21.1)		0.942 (0.820–1.083)	
Increased	4142 (67.4)	1345 (57.8)	987 (72.0)	419 (76.0)	1391 (73.4)		1.024 (0.896–1.171)	

Answers of “much increased” and “increased” have been merged; Answers of “decreased” and “much decreased” have been merged; IES-R: Impact of Event Scale–Revised; CI: confidence interval;

*p-value was based on Chi-square test;

** *p*-value was based on generalized linear model analysis.

A Chi-square analysis revealed significant association between IES-R categories with getting support from friends (*X*^2^ = 265.459; *p*<0.001), getting support from family members (*X*^2^ = 215.531; *p*<0.001), sharing feelings with family members (*X*^2^ = 340.216; *p*<0.001), sharing feelings with others (*X*^2^ = 450.398; *p*<0.001), and caring for family members’ feelings (*X*^2^ = 194.155; *p*<0.001). The multivariate regression analysis revealed that increased support from friends (Estimated rate ratio: 8.3%; *p*<0.001), and increased sharing feelings with others (Estimated rate ratio: 7.9%; *p*<0.001) were significantly associated with higher IES-R scores.

### 6. Mental health-related lifestyle changes

Table 6 displayed the association of IES-R scores with lifestyle indicators during the pandemic. About 41% of participants reported paying more attention to their mental health since the pandemic started. Additionally, over 40% of the participants reported spending more time to rest and relax. However, 41.8% of the participants reported spending less time exercising during the outbreak. A Chi-square analysis revealed significant association between IES-R categories with paying attention to mental health (*X*^2^ = 312.943; *p*<0.001), spending time to rest (*X*^2^ = 221.645; *p*<0.001), spending time to relax (*X*^2^ = 252.510; *p*<0.001), and spending time to exercise (*X*^2^ = 94.757; *p*<0.001). As expected, the multivariate regression analysis showed that decreased attention to mental health and decreased time spent to relax were significantly associated with higher IES-R scores (*p* = 0.001).

**Table 6:** Association of IES-R scores with lifestyle changes (*n* = 6142).

Variables	All	IES-R categories				*P*-value*	Rate Ratio (CI 95%)	*P*-value**
	n (%)							
	*n* = 6142	Normal	Mild	Moderate	Severe			
		n (%)	n (%)	n (%)	n (%)			
		*n* = 2325	*n* = 1371	*n* = 551	*n* = 1895			
Pay attention to mental health								
Decreased	544 (8.9)	125 (5.4)	120 (8.8)	55 (10.0)	244 (12.9)	<0.001	1	<0.001
Same as before	3111 (50.7)	1483 (63.8)	675 (49.2)	240 (43.6)	713 (37.6)		0.809 (0.736–0.891)	
Increased	2487 (40.5)	717 (30.8)	576 (42.0)	256 (46.5)	938 (49.5)		0.998 (0.905–1.100)	
Time spent to rest								
Decreased	1352 (22.0)	337 (14.5)	303 (22.1)	143 (26.0)	569 (30.0)	<0.001	1	0.039
Same as before	2102 (34.2)	1007 (43.3)	446 (32.5)	155 (28.1)	494 (26.1)		0.895 (0.816–0.982)	
Increased	2688 (43.8)	981 (42.2)	622 (45.4)	253 (45.9)	832 (43.9)		0.961 (0.872–1.059)	
Time spent to relax								
Decreased	1472 (24.0)	364 (15.7)	326 (23.8)	154 (27.9)	628 (33.1)	<0.001	1	0.001
Same as before	2169 (35.3)	1047 (45.0)	452 (33.0)	176 (31.9)	494 (26.1)		0.846 (0.773–0.926)	
Increased	2501 (40.7)	914 (39.3)	593 (43.3)	221 (40.1)	773 (40.8)		0.894 (0.812–0.984)	
Time spent to exercise								
Decreased	2569 (41.8)	838 (36.0)	569 (41.5)	242 (43.9)	920 (48.5)	<0.001	1	0.307
Same as before	2132 (34.7)	925 (39.8)	513 (37.4)	175 (31.8)	519 (27.4)		0.954 (0.896–1.015)	
Increased	1441 (23.5)	562 (24.2)	289 (21.1)	134 (24.3)	456 (24.1)		0.967 (0.903–1.036)	

Answers of “much increased” and “increased” have been merged; Answers of “decreased” and “much decreased” have been merged; IES-R: Impact of Event Scale–Revised; CI: confidence interval;

*p-value was based on Chi-square test;

** *p*-value was based on generalized linear model analysis.

## Discussion

This study aimed to investigate the impact of the COVID-19 outbreak on mental health and quality of life among residents of the MENA region. The survey was conducted after two months of lockdown measures implemented in the MENA region. Moreover, the pandemic is not over yet and it is rapidly expanding in many countries of the Middle East. Although many studies have examined the physiological effect of COVID-19, however, to our knowledge, this is the first large-scale study published investigating mental health and quality of life across eighteen countries in the MENA region.

Findings of this study suggested that participants from North Africa region were more likely to have higher stress scores compared to those residing in the Gulf and Levant regions. This study was conducted between May and mid of June 2020. During which, all countries included in the survey have already declared the state of emergency due to COVID-19 [34]. The number of confirmed cases by mid of June varied widely between MENA countries and ranged between 127 thousand cases in Saudi Arabia and 177 cases in Syrian Arab Republic [35]. Moreover, the number of deaths also varied in the same period and ranged between 972 in Saudi Arabia and 6 cases in Syrian Arab Republic [35]. Although, variation in the number of confirmed cases and deaths were observed between countries and sub-regions of the MENA, the greatest number of confirmed cases and deaths were reported in the Gulf region. All countries included in the study implemented strict measures between March 2020 and June 2020 or even longer [34]. This study might not reflect the diverse impact on the entire population from the MENA region considering the different stages of the pandemic in different countries.

The results of this study showed that about 40% of the participants in the MENA region had an IES-R score indicating moderate to severe disturbance due to the pandemic. Similarly, a study among Lebanese citizens has shown a rise of Post-traumatic Stress Disorder (PTSD) symptomatology during the fourth week of the COVID-19 quarantine [36]. Additionally, an online survey conducted in Saudi Arabia reported mild to moderate rates of anxiety among the general population and a significantly higher level of anxiety among married respondents [37]. In the current study females and participants aged 26–35 years were more likely to have higher stress scores. Likewise, a study in Saudi Arabia assessed the psychological impact of COVID-19 using the IES-R, and the Depression, Anxiety, and Stress Scale (DASS-21), and found that health care workers, students and females had higher levels of stress, anxiety and depression symptoms [32]. Our results also agree with recent studies from China and Italy which revealed that females are more vulnerable to stress compared to males, and that younger age groups had a higher tendency to be stimulated by the surrounding stressors [5, 38].

The biological, social and cognitive processes underlying gender differences in the susceptibility to psychological disorders have not yet been fully understood. However, some evidence indicates that fluctuations in ovarian hormone levels may be responsible for altered sensitivity to emotional stimuli among women [39]. Additionally, studies suggest that greater brainstem activation to threat stimuli may contribute to the greater prevalence of PTSD among women, and greater hippocampal activation in men may enhance their capacity for contextualizing fear-related stimuli [40, 41]. Telehealth services such as telephone counseling helplines, are useful to provide support to the vulnerable groups and is an appropriate tool for the delivery of mental health services [42]. Additionally, implementing community-based strategies to support psychologically vulnerable individuals during the COVID-19 pandemic is essential [18]. Likewise, awareness about self-relaxation and self-care measures for participants and their families can be encouraged to lessen social isolation [43].

The findings of this study indicated that during the pandemic, over one-third of the participants experienced increased stress related to work and financial matters, and over half of the participants had increased stress related to home matters. Similar trends were reported in a study among Egyptian adults, where 34.1% of participants reported an increase of stress from work, 55.7% had increased financial stress, and 62.7% had increased stress related to home matters [33]. Moreover, during the COVID-19 pandemic families were more likely to experience a lack of support from external sources (such as from schools or childcare settings), and financial strain, especially lower-income individuals and those who lost their jobs as a result of the pandemic [44]. Furthermore, families were affected by prolonged school closure, requiring online education support and uncertainty about examinations and enrolment arrangements [45]. With limited resources and barriers to providing assistance through welfare initiatives, governments and health care professionals must unite their efforts to protect high risk vulnerable people with additional support such as online peer group support sessions, psychoeducation, home-based relaxation techniques and stress management skills with online guidance [46].

Additionally, the study found that high school and college educated participants were more likely to have experienced an increased level of stress compared to those with higher education. Conflicting findings about the possible relationship between the level of education and stressful impact were reported in the literature. Some studies suggests that those with a higher level of education might practice better coping strategies and therefore report less stress score [47, 48]. Others proposed that highly educated people might be more stressed due to higher self-awareness and discernment of the pandemic severity [49, 50].

More than half of the participants in this study felt shock apprehension due to the pandemic, however they did not feel helpless as they reported paying more attention to their mental health and spending more time relaxing and resting during the pandemic. Additionally, the majority of participants reported getting increased support from family members as well as caring more about the feelings of family members during the pandemic. Such positive impacts on mental health may have helped the participants to cope with other negative impacts of the COVID-19 pandemic. The increased family support observed in this study was in line with previous studies from Egypt and China which demonstrated that family and friends were much valued in a time of crisis [22, 31, 33]. Researchers suggested that during quarantine, family members had the ability to spend more time together and were also more concerned about their health and family, while less so about leisure activities and friends [51]. On the other hand, the World Health Organization Europe member states have reported a 60% increase in emergency calls from women subjected to violence by their intimate partner during the pandemic [52]. Domestic violence reports in France have increased by 30% and domestic violence calls in Argentina have increased by 25% [53]. Similarly, in New York City the Police Department responded to a 10% increase in domestic violence reports during March 2020 compared to March 2019 [54]. Reasons could include job losses, rising alcohol based harm and drug use, stress and fear [52].

Unfortunately, about 42% of participants reported spending less time exercising during the outbreak. Recent evidence suggests that levels of physical activity were also negatively affected during quarantine [55–57]. It might be due to the widespread closure of sport facilities and parks, as well as complete lockdowns. Achieving minimum physical activity levels and reducing sedentary behavior during quarantine is a challenge and a necessity for everyone. A study investigating the influence of home confinement during the COVID-19 pandemic outbreak on lifestyle and mental wellbeing among Arab adults revealed that the mental wellbeing score was significantly higher among participants with medium to high physical activity levels [58]. Several studies have indicated the positive impact of physical activity as an effective therapy in support of mental and physical health [59, 60]. Moreover, physical activity and exercise were recommended as a therapy to fight against the mental and physical consequences of quarantine during the SARS and COVID-19 outbreaks [61, 62]. Home-based physical activity interventions are feasible, safe, and an effective way to increase physical activity among the general population [63]. Therefore, awareness about different types of home exercises and their benefits on mental health is essential [61, 64].

Limitations of this study include the use of self-reported questionnaire which might cause some respondent bias or misreporting of data; and the cross-sectional study design which provides only a snapshot of psychological responses at a particular point in time. Another potential limitation of this study was the use of snowballing sampling strategy, which is a non-probability sampling technique without adjusting for the population size of different countries. Also using an online survey limited the reach to non-social media users which led to less generalizable results. Moreover, information on the stressful impact due to political or economic status prior to the pandemic were not determined in the study. However, due to the time-sensitivity of the outbreak and with a strict quarantine measures in place, using an online survey allowed data collection from eighteen countries in the MENA region. It also guaranteed the anonymity of the participants, therefore reducing the social desirability bias. Another strength of this research project was conducting the survey in multiple languages, which allowed for wider distribution in the MENA region countries.

The current study identified females, younger age groups (26–35 years), people with school or college education and those residing in the North Africa region as high-risk groups to suffer from psychological distress. Additionally, recent studies also revealed that health care workers, students, people with history of medical problems, as well as those infected with COVID-19 and their family members are prone to psychological disorders [5, 32, 38, 50, 65]. Therefore, clinical interventions targeted towards vulnerable groups are needed to improve their mental health during the ongoing pandemic.

## Conclusion

This large-scale study across 18 countries, is the first study to our knowledge, investigating mental health and quality of life in the MENA region due to the COVID-19 pandemic. The findings of this study indicate that the COVID-19 pandemic was associated with mild psychological impact among adults in the MENA region. However, it also encouraged some positive impacts on family support and mental health awareness. There is a need to increase the awareness among the various media platforms about psychological challenges during pandemics and highlight the importance of seeking help and engaging in physical activity for the management of mental health disorders. Furthermore, an increase in awareness among the health care professionals in identifying and targeting the high-risk groups of the population who are at risk in developing mental health problems is vitally important.

Governments and policymakers must offer moral and financial support for low-income families and those who lost their jobs. Also, regulating working hours is needed to reduce the burden on individuals during the current pandemic. Future large-scale comparable studies among other age groups such as adolescents and children will help public health authorities shape their reactions and interventions in the future in response to similar crises.

## Supporting information

S1 File Statistical analysis plan. (PDF)

S2 File Data set. (SAV)
