# Supplementary material for: Impact of COVID-19 on mental health and quality of life: Is there any effect? A cross-sectional study of the MENA region
Source: PLoS One. 2021 Mar 25;16(3):e0249107. doi: 10.1371/journal.pone.0249107 (PMC7993788; doi:10.1371/journal.pone.0249107)
Supplement: S1 File — (PDF) [file pone.0249107.s001.pdf]

Statistical Analysis Plan

Date: 19 February 2021

Study title:	**Impact of COVID-19 on Mental Health and Quality of Life: Is There Any Effect? A Cross-Sectional Study of the MENA Region**	
Main investigator and corresponding author	**Ayesha S. Al Dhaheri**	**Signature:** **Date: 19 February 2021**
Main investigator	**Leila Cheikh Ismail**	**Signature:** **Date: 19 February 2021**
Co-investigator and biostatistician	**Mo'ath F. Bataineh**	**Signature:** **Date: 19 February 2021**
Co-investigator and biostatistician	**Maysm N. Mohamad**	**Signature:** **Date: 19 February 2021**

1. Objectives

To examine the impact of the COVID-19 outbreak on the mental health and quality of life among residents of the Middle East and North Africa region.

2. Sample size calculation

The minimum sample size (n=3246) was calculated using G*power software, version 3.1.9.4 (HHU, Germany) to detect a small effect size (0.10), with a power of 0.99, and alpha 0.05.

3. Inclusion criteria

- Age ≥ 18 years old
- Residing in one of the eighteen countries included in the study (Algeria, Bahrain, Egypt, Iraq, Jordan, Kuwait, Lebanon, Libya, Morocco, Oman, Palestine, Qatar, Republic of Yemen, Saudi Arabia, Sudan, Syria, Tunisia, and United Arab Emirates)
- Consenting to take part in the study

4. Measured variables retained for the analysis

Table 1: Sociodemographic

	Variables	Format	Description
1	Gender	Count	Gender: 1= Female; 2= Male
1	Age	Count	Age in years: 1=18-25 years; 2=26-35 years; 3=36-45 years; 4=>46 years
2	Marital status	Count	Marital status:1= Married; 2= Single; 3= Divorced; 4= Widowed
3	Education level	Count	Level of education: 1=≤ College diploma; 2= University degree; 3= Post-graduate degree
4	Employment status	Count	Type of employment: 1= Full-time; 2= Part-time; 3= Unemployed
5	Working/Studying from home	Count	Working or studying from home: 1= Yes; 2= No; 3= Not applicable
6	Region of residence	Count	Region of residence: 1= Gulf region, 2= Levant region; 3= North Africa region

Table 2: Impact of Event Scale–Revised (IES-R)

	Variables	Format	Description
1	**IES.1**<br>Any reminder brought back feelings about it.	Count	0=Not at all; 1=A little bit; 2=Moderately; 3= Quite a bit; 4=Extremely
2	**IES.2**<br>I had trouble staying asleep.	Count	0=Not at all; 1=A little bit; 2=Moderately; 3= Quite a bit; 4=Extremely
3	**IES.3**<br>Other things kept making me think about it.	Count	0=Not at all; 1=A little bit; 2=Moderately; 3= Quite a bit; 4=Extremely
4	**IES.4**<br>I felt irritable and angry.	Count	0=Not at all; 1=A little bit; 2=Moderately; 3= Quite a bit; 4=Extremely
5	**IES.5**<br>I avoided letting myself get upset when I thought about it or was reminded of it.	Count	0=Not at all; 1=A little bit; 2=Moderately; 3= Quite a bit; 4=Extremely
6	**IES.6**<br>I thought about it when I didn't mean to.	Count	0=Not at all; 1=A little bit; 2=Moderately; 3= Quite a bit; 4=Extremely

7	**IES.7**<br>I felt as if it hadn't happened or wasn't real.	Count	0=Not at all; 1=A little bit; 2=Moderately; 3= Quite a bit; 4=Extremely
8	**IES.8**<br>I stayed away from reminders of it.	Count	0=Not at all; 1=A little bit; 2=Moderately; 3= Quite a bit; 4=Extremely
9	**IES.9**<br>Pictures about it popped into my mind.	Count	0=Not at all; 1=A little bit; 2=Moderately; 3= Quite a bit; 4=Extremely
10	**IES.10**<br>I was jumpy and easily startled.	Count	0=Not at all; 1=A little bit; 2=Moderately; 3= Quite a bit; 4=Extremely
11	**IES.11**<br>I tried not to think about it.	Count	0=Not at all; 1=A little bit; 2=Moderately; 3= Quite a bit; 4=Extremely
12	**IES.12**<br>I was aware that I still had a lot of feelings about it, but I didn't deal with them.	Count	0=Not at all; 1=A little bit; 2=Moderately; 3= Quite a bit; 4=Extremely
13	**IES.13**<br>My feelings about it were kind of numb.	Count	0=Not at all; 1=A little bit; 2=Moderately; 3= Quite a bit; 4=Extremely
14	**IES.14**<br>I found myself acting or feeling like I was back at that time.	Count	0=Not at all; 1=A little bit; 2=Moderately; 3= Quite a bit; 4=Extremely
15	**IES.15**<br>I had trouble falling asleep.	Count	0=Not at all; 1=A little bit; 2=Moderately; 3= Quite a bit; 4=Extremely
16	**IES.16**<br>I had waves of strong feelings about it.	Count	0=Not at all; 1=A little bit; 2=Moderately; 3= Quite a bit; 4=Extremely
17	**IES.17**<br>I tried to remove it from my memory.	Count	0=Not at all; 1=A little bit; 2=Moderately; 3= Quite a bit; 4=Extremely
18	**IES.18**<br>I had trouble concentrating.	Count	0=Not at all; 1=A little bit; 2=Moderately; 3= Quite a bit; 4=Extremely
19	**IES.19**<br>Reminders of it caused me to have physical reactions, such as sweating, trouble breathing, nausea, or a pounding heart.	Count	0=Not at all; 1=A little bit; 2=Moderately; 3= Quite a bit; 4=Extremely

20	**IES.20**<br>I had dreams about it.	Count	0=Not at all; 1=A little bit; 2=Moderately; 3= Quite a bit; 4=Extremely
21	**IES.21**<br>I felt watchful and on-guard.	Count	0=Not at all; 1=A little bit; 2=Moderately; 3= Quite a bit; 4=Extremely
22	**IES.22**<br>I tried not to talk about it.	Count	0=Not at all; 1=A little bit; 2=Moderately; 3= Quite a bit; 4=Extremely

Table 3: IES-R scores with sociodemographic factors

	**Variables**	**Format**	**Description**
1	Gender	Count	Gender: 1= Female; 2= Male
1	Age	Count	Age in years: 1=18-25 years; 2=26-35 years; 3=36-45 years; 4=>46 years
2	Marital status	Count	Marital status:1= Married; 2= Single; 3= Divorced; 4= Widowed
3	Education level	Count	Level of education: 1=≤ College diploma; 2= University degree; 3= Post-graduate degree
4	Employment status	Count	Type of employment: 1= Full-time; 2= Part-time; 3= Unemployed
5	Working/Studying from home	Count	Working or studying from home: 1= Yes; 2= No; 3= Not applicable
6	Region of residence	Count	Region of residence: 1= Gulf region, 2= Levant region; 3= North Africa region
7	**IES.1**<br>Any reminder brought back feelings about it.	Count	0=Not at all; 1=A little bit; 2=Moderately; 3= Quite a bit; 4=Extremely
8	**IES.2**<br>I had trouble staying asleep.	Count	0=Not at all; 1=A little bit; 2=Moderately; 3= Quite a bit; 4=Extremely
9	**IES.3**<br>Other things kept making me think about it.	Count	0=Not at all; 1=A little bit; 2=Moderately; 3= Quite a bit; 4=Extremely
10	**IES.4**<br>I felt irritable and angry.	Count	0=Not at all; 1=A little bit; 2=Moderately; 3= Quite a bit; 4=Extremely
11	**IES.5**<br>I avoided letting myself get upset when I thought about it or was reminded of it.	Count	0=Not at all; 1=A little bit; 2=Moderately; 3= Quite a bit; 4=Extremely

12	**IES.6**<br>I thought about it when I didn't mean to.	Count	0=Not at all; 1=A little bit; 2=Moderately; 3= Quite a bit; 4=Extremely
13	**IES.7**<br>I felt as if it hadn't happened or wasn't real.	Count	0=Not at all; 1=A little bit; 2=Moderately; 3= Quite a bit; 4=Extremely
14	**IES.8**<br>I stayed away from reminders of it.	Count	0=Not at all; 1=A little bit; 2=Moderately; 3= Quite a bit; 4=Extremely
15	**IES.9**<br>Pictures about it popped into my mind.	Count	0=Not at all; 1=A little bit; 2=Moderately; 3= Quite a bit; 4=Extremely
16	**IES.10**<br>I was jumpy and easily startled.	Count	0=Not at all; 1=A little bit; 2=Moderately; 3= Quite a bit; 4=Extremely
17	**IES.11**<br>I tried not to think about it.	Count	0=Not at all; 1=A little bit; 2=Moderately; 3= Quite a bit; 4=Extremely
18	**IES.12**<br>I was aware that I still had a lot of feelings about it, but I didn't deal with them.	Count	0=Not at all; 1=A little bit; 2=Moderately; 3= Quite a bit; 4=Extremely
19	**IES.13**<br>My feelings about it were kind of numb.	Count	0=Not at all; 1=A little bit; 2=Moderately; 3= Quite a bit; 4=Extremely
20	**IES.14**<br>I found myself acting or feeling like I was back at that time.	Count	0=Not at all; 1=A little bit; 2=Moderately; 3= Quite a bit; 4=Extremely
21	**IES.15**<br>I had trouble falling asleep.	Count	0=Not at all; 1=A little bit; 2=Moderately; 3= Quite a bit; 4=Extremely
22	**IES.16**<br>I had waves of strong feelings about it.	Count	0=Not at all; 1=A little bit; 2=Moderately; 3= Quite a bit; 4=Extremely
23	**IES.17**<br>I tried to remove it from my memory.	Count	0=Not at all; 1=A little bit; 2=Moderately; 3= Quite a bit; 4=Extremely
24	**IES.18**<br>I had trouble concentrating.	Count	0=Not at all; 1=A little bit; 2=Moderately; 3= Quite a bit; 4=Extremely
25	**IES.19**<br>Reminders of it caused me to have physical reactions, such as sweating, trouble	Count	0=Not at all; 1=A little bit; 2=Moderately; 3= Quite a bit; 4=Extremely

	breathing, nausea, or a pounding heart.		
26	**IES.20**<br>I had dreams about it.	Count	0=Not at all; 1=A little bit; 2=Moderately; 3= Quite a bit; 4=Extremely
27	**IES.21**<br>I felt watchful and on-guard.	Count	0=Not at all; 1=A little bit; 2=Moderately; 3= Quite a bit; 4=Extremely
28	**IES.22**<br>I tried not to talk about it.	Count	0=Not at all; 1=A little bit; 2=Moderately; 3= Quite a bit; 4=Extremely

Table 4: IES-R scores with negative mental health indicators

	**Variables**	**Format**	**Description**
1	**Negative.1**<br>Are you experiencing increased stress from work?	Count	1=Much decreased; 2=decreased; 3= same as before; 4=increased; 5= much increased
2	**Negative.2**<br>Are you experiencing increased financial stress from the outbreak?	Count	1=Much decreased; 2=decreased; 3= same as before; 4=increased; 5= much increased
3	**Negative.3**<br>Are you experiencing increased stress from staying at home?	Count	1=Much decreased; 2=decreased; 3= same as before; 4=increased; 5= much increased
4	**Negative.4**<br>Have you felt horrified due to COVID-19?	Count	1=Much decreased; 2=decreased; 3= same as before; 4=increased; 5= much increased
5	**Negative.5**<br>Have you felt apprehensive due to COVID-19?	Count	1=Much decreased; 2=decreased; 3= same as before; 4=increased; 5= much increased
6	**Negative.6**<br>Have you felt helpless due to COVID-19?	Count	1=Much decreased; 2=decreased; 3= same as before; 4=increased; 5= much increased
7	**IES.1**<br>Any reminder brought back feelings about it.	Count	0=Not at all; 1=A little bit; 2=Moderately; 3= Quite a bit; 4=Extremely
8	**IES.2**<br>I had trouble staying asleep.	Count	0=Not at all; 1=A little bit; 2=Moderately; 3= Quite a bit; 4=Extremely
9	**IES.3**<br>Other things kept making me think about it.	Count	0=Not at all; 1=A little bit; 2=Moderately; 3= Quite a bit; 4=Extremely

10	**IES.4**<br>I felt irritable and angry.	Count	0=Not at all; 1=A little bit; 2=Moderately; 3= Quite a bit; 4=Extremely
11	**IES.5**<br>I avoided letting myself get upset when I thought about it or was reminded of it.	Count	0=Not at all; 1=A little bit; 2=Moderately; 3= Quite a bit; 4=Extremely
12	**IES.6**<br>I thought about it when I didn't mean to.	Count	0=Not at all; 1=A little bit; 2=Moderately; 3= Quite a bit; 4=Extremely
13	**IES.7**<br>I felt as if it hadn't happened or wasn't real.	Count	0=Not at all; 1=A little bit; 2=Moderately; 3= Quite a bit; 4=Extremely
14	**IES.8**<br>I stayed away from reminders of it.	Count	0=Not at all; 1=A little bit; 2=Moderately; 3= Quite a bit; 4=Extremely
15	**IES.9**<br>Pictures about it popped into my mind.	Count	0=Not at all; 1=A little bit; 2=Moderately; 3= Quite a bit; 4=Extremely
16	**IES.10**<br>I was jumpy and easily startled.	Count	0=Not at all; 1=A little bit; 2=Moderately; 3= Quite a bit; 4=Extremely
17	**IES.11**<br>I tried not to think about it.	Count	0=Not at all; 1=A little bit; 2=Moderately; 3= Quite a bit; 4=Extremely
18	**IES.12**<br>I was aware that I still had a lot of feelings about it, but I didn't deal with them.	Count	0=Not at all; 1=A little bit; 2=Moderately; 3= Quite a bit; 4=Extremely
19	**IES.13**<br>My feelings about it were kind of numb.	Count	0=Not at all; 1=A little bit; 2=Moderately; 3= Quite a bit; 4=Extremely
20	**IES.14**<br>I found myself acting or feeling like I was back at that time.	Count	0=Not at all; 1=A little bit; 2=Moderately; 3= Quite a bit; 4=Extremely
21	**IES.15**<br>I had trouble falling asleep.	Count	0=Not at all; 1=A little bit; 2=Moderately; 3= Quite a bit; 4=Extremely
22	**IES.16**<br>I had waves of strong feelings about it.	Count	0=Not at all; 1=A little bit; 2=Moderately; 3= Quite a bit; 4=Extremely
23	**IES.17**<br>I tried to remove it from my memory.	Count	0=Not at all; 1=A little bit; 2=Moderately; 3= Quite a bit; 4=Extremely

24	**IES.18**<br>I had trouble concentrating.	Count	0=Not at all; 1=A little bit; 2=Moderately; 3= Quite a bit; 4=Extremely
25	**IES.19**<br>Reminders of it caused me to have physical reactions, such as sweating, trouble breathing, nausea, or a pounding heart.	Count	0=Not at all; 1=A little bit; 2=Moderately; 3= Quite a bit; 4=Extremely
26	**IES.20**<br>I had dreams about it.	Count	0=Not at all; 1=A little bit; 2=Moderately; 3= Quite a bit; 4=Extremely
27	**IES.21**<br>I felt watchful and on-guard.	Count	0=Not at all; 1=A little bit; 2=Moderately; 3= Quite a bit; 4=Extremely
28	**IES.22**<br>I tried not to talk about it.	Count	0=Not at all; 1=A little bit; 2=Moderately; 3= Quite a bit; 4=Extremely

Table 5: IES-R scores with impact on family and social support

	**Variables**	**Format**	**Description**
1	**Support.1**<br>Are you getting support from friends?	Count	1=Much decreased; 2=decreased; 3= same as before; 4=increased; 5= much increased
2	**Support.2**<br>Are you getting support from family members?	Count	1=Much decreased; 2=decreased; 3= same as before; 4=increased; 5= much increased
3	**Support.3**<br>Are you sharing feelings with family members?	Count	1=Much decreased; 2=decreased; 3= same as before; 4=increased; 5= much increased
4	**Support.4**<br>Are you sharing feelings with others when feeling blue?	Count	1=Much decreased; 2=decreased; 3= same as before; 4=increased; 5= much increased
5	**Support.5**<br>Are you caring for family members' feelings?	Count	1=Much decreased; 2=decreased; 3= same as before; 4=increased; 5= much increased
6	**IES.1**<br>Any reminder brought back feelings about it.	Count	0=Not at all; 1=A little bit; 2=Moderately; 3= Quite a bit; 4=Extremely
7	**IES.2**<br>I had trouble staying asleep.	Count	0=Not at all; 1=A little bit; 2=Moderately; 3= Quite a bit; 4=Extremely

8	**IES.3**<br>Other things kept making me think about it.	Count	0=Not at all; 1=A little bit; 2=Moderately; 3= Quite a bit; 4=Extremely
9	**IES.4**<br>I felt irritable and angry.	Count	0=Not at all; 1=A little bit; 2=Moderately; 3= Quite a bit; 4=Extremely
10	**IES.5**<br>I avoided letting myself get upset when I thought about it or was reminded of it.	Count	0=Not at all; 1=A little bit; 2=Moderately; 3= Quite a bit; 4=Extremely
11	**IES.6**<br>I thought about it when I didn't mean to.	Count	0=Not at all; 1=A little bit; 2=Moderately; 3= Quite a bit; 4=Extremely
12	**IES.7**<br>I felt as if it hadn't happened or wasn't real.	Count	0=Not at all; 1=A little bit; 2=Moderately; 3= Quite a bit; 4=Extremely
13	**IES.8**<br>I stayed away from reminders of it.	Count	0=Not at all; 1=A little bit; 2=Moderately; 3= Quite a bit; 4=Extremely
14	**IES.9**<br>Pictures about it popped into my mind.	Count	0=Not at all; 1=A little bit; 2=Moderately; 3= Quite a bit; 4=Extremely
15	**IES.10**<br>I was jumpy and easily startled.	Count	0=Not at all; 1=A little bit; 2=Moderately; 3= Quite a bit; 4=Extremely
16	**IES.11**<br>I tried not to think about it.	Count	0=Not at all; 1=A little bit; 2=Moderately; 3= Quite a bit; 4=Extremely
17	**IES.12**<br>I was aware that I still had a lot of feelings about it, but I didn't deal with them.	Count	0=Not at all; 1=A little bit; 2=Moderately; 3= Quite a bit; 4=Extremely
18	**IES.13**<br>My feelings about it were kind of numb.	Count	0=Not at all; 1=A little bit; 2=Moderately; 3= Quite a bit; 4=Extremely
19	**IES.14**<br>I found myself acting or feeling like I was back at that time.	Count	0=Not at all; 1=A little bit; 2=Moderately; 3= Quite a bit; 4=Extremely
20	**IES.15**<br>I had trouble falling asleep.	Count	0=Not at all; 1=A little bit; 2=Moderately; 3= Quite a bit; 4=Extremely
21	**IES.16**<br>I had waves of strong feelings about it.	Count	0=Not at all; 1=A little bit; 2=Moderately; 3= Quite a bit; 4=Extremely

22	**IES.17**<br>I tried to remove it from my memory.	Count	0=Not at all; 1=A little bit; 2=Moderately; 3= Quite a bit; 4=Extremely
23	**IES.18**<br>I had trouble concentrating.	Count	0=Not at all; 1=A little bit; 2=Moderately; 3= Quite a bit; 4=Extremely
24	**IES.19**<br>Reminders of it caused me to have physical reactions, such as sweating, trouble breathing, nausea, or a pounding heart.	Count	0=Not at all; 1=A little bit; 2=Moderately; 3= Quite a bit; 4=Extremely
25	**IES.20**<br>I had dreams about it.	Count	0=Not at all; 1=A little bit; 2=Moderately; 3= Quite a bit; 4=Extremely
26	**IES.21**<br>I felt watchful and on-guard.	Count	0=Not at all; 1=A little bit; 2=Moderately; 3= Quite a bit; 4=Extremely
27	**IES.22**<br>I tried not to talk about it.	Count	0=Not at all; 1=A little bit; 2=Moderately; 3= Quite a bit; 4=Extremely

Table 6: IES-R scores with lifestyle changes

	**Variables**	**Format**	**Description**
1	**Lifestyle.1**<br>Are you paying attention to your mental health?	Count	1=Much decreased; 2=decreased; 3= same as before; 4=increased; 5= much increased
2	**Lifestyle.2**<br>Do you spend enough time to rest?	Count	1=Much decreased; 2=decreased; 3= same as before; 4=increased; 5= much increased
3	**Lifestyle.3**<br>Do you spend enough time to relax?	Count	1=Much decreased; 2=decreased; 3= same as before; 4=increased; 5= much increased
4	**Lifestyle.4**<br>Do you spend enough time to exercise?	Count	1=Much decreased; 2=decreased; 3= same as before; 4=increased; 5= much increased
5	**IES.1**<br>Any reminder brought back feelings about it.	Count	0=Not at all; 1=A little bit; 2=Moderately; 3= Quite a bit; 4=Extremely
6	**IES.2**<br>I had trouble staying asleep.	Count	0=Not at all; 1=A little bit; 2=Moderately; 3= Quite a bit; 4=Extremely
7	**IES.3**<br>Other things kept making me think about it.	Count	0=Not at all; 1=A little bit; 2=Moderately; 3= Quite a bit; 4=Extremely

8	**IES.4**<br>I felt irritable and angry.	Count	0=Not at all; 1=A little bit; 2=Moderately; 3= Quite a bit; 4=Extremely
9	**IES.5**<br>I avoided letting myself get upset when I thought about it or was reminded of it.	Count	0=Not at all; 1=A little bit; 2=Moderately; 3= Quite a bit; 4=Extremely
10	**IES.6**<br>I thought about it when I didn't mean to.	Count	0=Not at all; 1=A little bit; 2=Moderately; 3= Quite a bit; 4=Extremely
11	**IES.7**<br>I felt as if it hadn't happened or wasn't real.	Count	0=Not at all; 1=A little bit; 2=Moderately; 3= Quite a bit; 4=Extremely
12	**IES.8**<br>I stayed away from reminders of it.	Count	0=Not at all; 1=A little bit; 2=Moderately; 3= Quite a bit; 4=Extremely
13	**IES.9**<br>Pictures about it popped into my mind.	Count	0=Not at all; 1=A little bit; 2=Moderately; 3= Quite a bit; 4=Extremely
14	**IES.10**<br>I was jumpy and easily startled.	Count	0=Not at all; 1=A little bit; 2=Moderately; 3= Quite a bit; 4=Extremely
15	**IES.11**<br>I tried not to think about it.	Count	0=Not at all; 1=A little bit; 2=Moderately; 3= Quite a bit; 4=Extremely
16	**IES.12**<br>I was aware that I still had a lot of feelings about it, but I didn't deal with them.	Count	0=Not at all; 1=A little bit; 2=Moderately; 3= Quite a bit; 4=Extremely
17	**IES.13**<br>My feelings about it were kind of numb.	Count	0=Not at all; 1=A little bit; 2=Moderately; 3= Quite a bit; 4=Extremely
18	**IES.14**<br>I found myself acting or feeling like I was back at that time.	Count	0=Not at all; 1=A little bit; 2=Moderately; 3= Quite a bit; 4=Extremely
19	**IES.15**<br>I had trouble falling asleep.	Count	0=Not at all; 1=A little bit; 2=Moderately; 3= Quite a bit; 4=Extremely
20	**IES.16**<br>I had waves of strong feelings about it.	Count	0=Not at all; 1=A little bit; 2=Moderately; 3= Quite a bit; 4=Extremely
21	**IES.17**<br>I tried to remove it from my memory.	Count	0=Not at all; 1=A little bit; 2=Moderately; 3= Quite a bit; 4=Extremely

22	**IES.18**<br>I had trouble concentrating.	Count	0=Not at all; 1=A little bit;<br>2=Moderately; 3= Quite a bit;<br>4=Extremely
23	**IES.19**<br>Reminders of it caused me to have physical reactions, such as sweating, trouble breathing, nausea, or a pounding heart.	Count	0=Not at all; 1=A little bit;<br>2=Moderately; 3= Quite a bit;<br>4=Extremely
24	**IES.20**<br>I had dreams about it.	Count	0=Not at all; 1=A little bit;<br>2=Moderately; 3= Quite a bit;<br>4=Extremely
25	**IES.21**<br>I felt watchful and on-guard.	Count	0=Not at all; 1=A little bit;<br>2=Moderately; 3= Quite a bit;<br>4=Extremely
26	**IES.22**<br>I tried not to talk about it.	Count	0=Not at all; 1=A little bit;<br>2=Moderately; 3= Quite a bit;<br>4=Extremely

5. Statistical methods

Normality of data was tested with the use of Kolmogorov-Smirnov test of normality. Descriptive statistics for the sociodemographic characteristics were reported as numbers and percentages. The IES-R total and subscale scores were presented as Median and Interquartile Range (IQR). A Chi-square ($\chi 2$) test was used to determine the association between IES-R categories (normal, mild, moderate, and severe) with categorical variables. A non-parametric Kruskal-Wallis H test was used to determine differences in IES-R, intrusion, avoidance, and hyperarousal scores between different regions. Followed by post-hoc pairwise comparisons with Bonferroni adjustment. A generalized linear model based on negative binomial distribution was used to assess the confounding effects of sociodemographic factors, negative mental health impact factors, social and family support indicators, and lifestyle factors on continuous IES-R total score. Variables included in the final model were selected using univariant general linear analysis and only factors with a cut-off value of $p<0.2$ were selected. A p-value <0.05 was considered to be statistically significant.

6. System and Software

All analyses were performed using the Statistical Package for the Social Sciences (SPSS) Version 26.0 (IBM, Chicago, IL, USA) running macOS Big Sur Version 11.2.1. Sample size was calculated using G*power software, version 3.1.9.4 (HHU, Germany).
